# What do mothers think about their antenatal classes? A mixed-method study in Switzerland

**DOI:** 10.1186/s12884-023-06049-8

**Published:** 2023-10-19

**Authors:** Valérie Avignon, Laurent Gaucher, David Baud, Hélène Legardeur, Corinne Dupont, Antje Horsch

**Affiliations:** 1 Department Woman-Mother-Child, Obstetric Service, Lausanne University Hospital (CHUV) and University of Lausanne, Avenue Pierre-Decker 2, Lausanne, 1011 Switzerland; 2 Research on Healthcare Performance RESHAPE, INSERM U1290, Université Claude Bernard Lyon 1, Lyon, 69008 France; 3 Institute of Higher Education and Research in Healthcare-IUFRS, University of Lausanne, Lausanne University Hospital, Route de La Corniche 10 - Bâtiment Proline, Lausanne, CH-1010 Switzerland; 4 Geneva School of Health Sciences, HES-SO University of Applied Sciences and Arts, Geneva, Western Switzerland 1206 Switzerland

**Keywords:** Prenatal education, Patient satisfaction, Maternal health services, Pregnant mothers, Delivery, Obstetric

## Abstract

**Problem:**

Research so far has evaluated the effect of antenatal classes, but few studies have investigated its usefulness from the perspective of mothers after birth.

**Background:**

Antenatal classes evolved from pain management to a mother-centred approach, including birth plans and parenting education. Evaluating the perception of the usefulness of these classes is important to meet mother’s needs. However, so far, research on the mothers’ perception of the usefulness of these classes is sparse, particularly when measured after childbirth. Given that antenatal classes are considered as adult education, it is necessary to carry out this evaluation after mothers have had an opportunity to apply some of the competences they acquired during the antenatal classes during their childbirth.

**Aim:**

This study investigated mothers’ satisfaction and perceived usefulness of antenatal classes provided within a university hospital in Switzerland, as assessed in the postpartum period.

**Methods:**

Primiparous mothers who gave birth at a Swiss university hospital from January 2018 to September 2020 were contacted. Those who had attended the hospital’s antenatal classes were invited to complete a questionnaire consisting of a quantitative and qualitative part about usefulness and satisfaction about antenatal classes. Quantitative data were analysed using both descriptive and inferential statistics. Qualitative data were analysed using thematic analysis.

**Findings:**

Among the 259 mothers who answered, 61% (*n* = 158) were globally satisfied with the antenatal classes and 56.2% (*n* = 145) found the sessions useful in general. However, looking at the utility score of each theme, none of them achieved a score of usefulness above 44%. The timing of some of these sessions was questioned. Some mothers regretted the lack of accurate information, especially on labour complications and postnatal care.

**Discussion:**

Antenatal classes were valued for their peer support. However, in their salutogenic vision of empowerment, they did not address the complications of childbirth, even though this was what some mothers needed. Furthermore, these classes could also be more oriented towards the postpartum period, as requested by some mothers.

**Conclusion:**

Revising antenatal classes to fit mothers’ needs could lead to greater satisfaction and thus a better impact on the well-being of mothers and their families.

**Supplementary Information:**

The online version contains supplementary material available at 10.1186/s12884-023-06049-8.

## Background

Antenatal classes (AC) were developed in the 1950s on the assumption that pain is not inherent to the birth process but that fear of childbirth generates tension, which in turn leads to pain [1]. The concepts of fearless childbirth (Dick-Read; 1890–1959) or painless childbirth strongly inspired by Pavlov’s theories (Lamaze; 1891–1957) stem from this principle [1, 2]. Painless childbirth, or obstetrical psychoprophylaxis, spread rapidly in Europe [2] and became commonplace in the 1970s [1]. Profound changes in AC in the 1980s [3] due to the development of feminism and the reduction of postnatal hospitalisation, led to a greater involvement of partners to share tasks with the newborn and support the mother at home. On the other hand, medicalisation of childbirth, including the development of epidurals, changed the AC’s content [3]. Pain control was no longer the main objective and new mother-centred approaches were developed, such as Leboyer’s non-violent childbirth, sophrology, yoga, prenatal singing, haptonomy, and aquanatal sessions [1].

Since the beginning of the 2000s, the psychoprophylactic approach (mental and physical training) received increased interest [4]. Theoretical knowledge about pregnancy and childbirth, breathing techniques, postural work, and relaxation remain the base of AC [5]. However, new AC goals are to enable the woman to develop her own birth plan, considering her preferences, emotions, and perceptions, as well as her life context [6]. Antenatal classes are now widely implemented in the Western World [7]. With a wide range of goals and modalities, the theoretical bases are linked to Dick-Read’s and Lamaze’s approaches, enhanced by novel theories like “Active Birth” and “Hypnobirthing” [7].

The evaluation of antenatal classes is scarce [4, 8] and mainly focuses on mothers’ knowledge, obstetrical, neonatal or psychological outcomes rather than on satisfaction [9]. However, exploring patient satisfaction, i.e., if the care approach fulfilled the patients’ expectations [10], is a key measure of healthcare quality [11]. However, evaluating it after the completion of the courses but before birth means only assessing what mothers think about the sessions and what they think they could do without taking into account what they were, really, able to do during birth and as new parents [12]. Several quantitative and qualitative studies explored the satisfaction with and perceived usefulness of AC after birth [13–16]. The poor quality of studies [8] and a lack of standardization and guidelines regarding the content and modalities of AC make it difficult to generalise the results [7]. However, antenatal classes seem to be necessary and should be proposed to all primiparous women [8]. Moreover, so far, no evaluation of mothers’ satisfaction and/or perceived usefulness of AC in Switzerland, after the birth, was available.

Therefore, we aimed to conduct a quantitative and qualitative evaluation of antenatal classes offered by our University Hospital from the mothers’ perspective, based on the Lamaze theoretical model.

## Methods

A mixed-method study analysing quantitative and qualitative sections of a satisfaction questionnaire was conducted. Quantitative and qualitative approaches are complementary, collected at the same time, and analysed a ccording to a convergent parallel method [17]. The questionnaire was part of a larger cross-sectional study investigating relationships between AC attendance, birth satisfaction, and symptoms of post-traumatic stress disorder [18].

### Setting

The Lausanne University Hospital (CHUV, Switzerland), realize approximately 3200 births per year. Pregnancies are monitored by a gynaecologist in the community or by a gynaecologist and/or midwife in the hospital During birth, one-to-one midwifery care is organised. After the birth, the hospital stay varies in general from 2 to 5 days. Following discharge, independent midwives follow up 93.8% of women at home, with an average of 7.6 home visits [19].

The CHUV proposes four different formats of AC: psychoprophylactic sessions, aqua natal sessions, antenatal exercise classes, and short antenatal sessions. Mothers also have the option of attending AC outside the hospital. AC are refunded by Swiss health insurances up to an amount of 150 Swiss francs per session. The session fees of the short antenatal sessions are based on this amount, enabling all pregnant women to sign up to AC.

The CHUV AC sessions, based on the Lamaze theoretical model, focus on coping strategies by emphasizing a pro-active problem-solving approach. They were designed to increase mothers’ self-confidence for childbirth and parenting by using case examples, allowing mothers and couples to project themselves into childbirth. Except for short AC sessions, programs comprise five two-hour sessions before birth (32 to 36 weeks of gestation), and one at 6 weeks after birth. Practical exercises are proposed in different formats (antenatal exercise classes, aqua natal sessions or psychoprophylactic sessions) including posture correction exercises, relaxation, and breathing techniques (see Supplementary materials Table S1). The time dedicated to the practical part is greater in antenatal exercise classes and aquanatal sessions, which makes them particularly popular with multiparous mothers. The short antenatal sessions are limited to two-hour theoretical sessions on childbirth or breastfeeding, without practical exercises. A previous publication highlighted that the AC attendees are generally higher educated and from a more privileged socio-economic background [18].

### Study procedure and participants

All primiparous mothers over 18 years, who gave birth to a single, live, term baby (≥ 37 weeks of gestational age) at the hospital between January 2018 to September 2020, were contacted by Short Message Services (SMS) from June 2020 to December 2020, with three reminders. This SMS invited them to complete a questionnaire via an internet link (see [18] for more details). Mothers indicated in the questionnaire whether they had attended AC and if so, where. Only mothers who attended CHUV antenatal classes were included in the present study.

### Measures

The quantitative part consisted of six items related to satisfaction (fulfilment of a need) and usefulness (relevance of the information to the context) of overall and different parts of the AC (pregnancy, childbirth, newborn’s and mother’s needs). The last author developed the questionnaire for another study [20] and adapted it to the childbirth context for this study. Each question used a five-point Likert scale ranging from “1 = Not at all satisfied” or “1 = Not at all helpful” to “5 = Extremely satisfied” or “5 = Extremely helpful”. A question about the timing of AC was answered with a three-point Likert scale: “too early”, “adequate” or “too late”. Mothers were also asked if they would recommend these AC to a friend.

The qualitative part consisted of a free text space available after each quantitative question, allowing mothers to explain their evaluation but also to express their feelings about each part of the AC. Moreover, three open-ended questions were asked: two about the three most helpful or difficult things regarding AC and another about the topics that mothers would have liked to have discussed and that were not addressed (See Supplementary material S3).

Demographic information (citizenship, civil status, educational background) and was collected via self-report questionnaires. Medical data (maternal age at birth, induction of labour, oxytocin augmentation, analgesia, mode of birth, admission in NICU) were extracted from medical records.

### Data analysis

This study consisted of two different but concurrent phases [17]. In this design, quantitative and qualitative data were collected together by questionnaire. The data were separately analysed and then merged, allowing qualitative data to support quantitative results [17].

Quantitative analyses were conducted with SPSS® (Statistical Package for Social Sciences, IBM®, version 27.0). Descriptive statistics for obstetric and neonatal outcomes, as well as for satisfaction and usefulness of the sessions were done. Bi-variate correlations were carried out to examine whether satisfaction and usefulness of the sessions were related to the number of sessions attended or the time between the childbirth and the completion of the questionnaire. Some demographic data and obstetrical data were missing (see Table 1). No missing data were replaced.

**Table 1:** Sociodemographic sample characteristics, obstetric and neonatal outcomes of mothers who attended antenatal classes (*n* = 289)

**Characteristics and obstetric and neonatal outcomes**	**CHUV Antenatal classes**
Questionnaire completion time since birth (days), M ± SD	467.40 ± 210.28
Maternal age at birth (years), M ± SD (no missing data)	32.8 ± 4.13
Country of origin, n (%)	(no missing data)
Switzerland	169 (58.5%)
European Union (UE)	105 (36.3%)
Europe except UE	7 (2.4%)
Americas	4 (1.4%)
Other countries	4 (1.4%)
Civil Status, n (%)	(One missing data)
Single/ Separate/Divorced/Widow	93 (32.3%)
Married / In common-law	195 (67.7%)
Educational background, n (%)	(One missing data)
Primary education/ Secondary education or other level	8 (2.8%)
Apprenticeship	55 (19.1%)
Higher secondary education	13 (4.5%)
University or higher education	212 (73.6%)
Mode of birth, n (%)	(no missing data)
Elective caesarean	16 (5.5%)
Emergency caesarean	44 (15.2%)
Forceps	35 (12.1%)
Vacuum extraction	20 (6.9%)
Spontaneous birth	174 (60.2%)
Induction of labour, n (%) (40 missing data)	94 (37.8%)
Oxytocin augmentation of labour, n (%) (40 missing data)	45 (17.0%)
Analgesia, n (%)	(no missing data)
None	12 (4.2%)
Local anaesthesia, Pudendal Nerve Block, EMONO (Nitrous Oxide/Oxygen 50%/50%)	49 (17.0%)
Epidural	195 (67.5%)
Spinal anaesthesia	32 (11.1%)
General anaesthesia	1 (0.3%)
NICU admission	24 (8.3%)

*M* ± *SD* Mean ± Standard Deviation, *NICU* Neonatal Intensive Care Unit

Qualitative analyses, conducted by the first author, used a hybrid approach of qualitative methods of thematic analysis [21]. It consisted firstly of a deductive approach using an a-priori template of codes based on the domains addressed in the questionnaire (Overall comment, Pregnancy, Childbirth, New-borns’ and Mothers’ needs) according to the approach of Crabtree and Miller [22]. Secondly, according to the methodology describe by Boyatzis [23], a data-driven inductive approach was implemented to isolate the themes within the pre-defined domains. This complementary approach allowed new themes and sub-themes to emerge within the domains induced by the structure of the questionnaire and thus provided additional data in the analysis of mothers’ satisfaction with their AC.

### Ethical issues

This project was developed in accordance with the rules and regulations in relation to research on human beings in force in Switzerland (risk category A). Each woman had to electronically sign an informed e-consent before participating in the study. The Ethical Committee of the Canton de Vaud approved the study protocol (n°2019–02228).

## Results

**Fig. 1:**
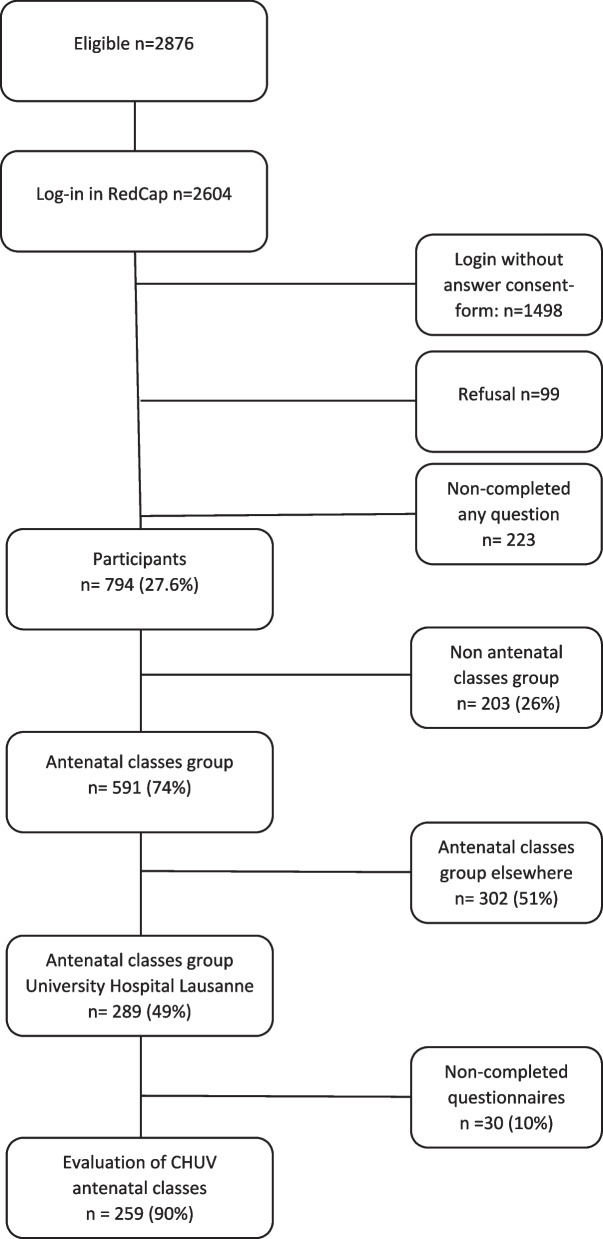
Study flow chart

Of the 2876 eligible mothers contacted in the main study, 289 mothers (10%) were involved in the AC at the CHUV (Fig. 1). Respondents had a mean age of 32.8 years, were mostly Swiss (58.5%; *n* = 169), in a couple relationship (67.7%; *n* = 195), and had a strong educational background (73.6%; *n* = 212). Participants mainly had a vaginal birth (79.2%; *n* = 229), with epidural analgesia (58.5%; *n* = 169), and gave birth to a healthy newborn (91.7%; *n* = 65; Table 1).

Among the 289 study participants, 89% had followed the psychoprophylactic sessions, 6% the aqua natal classes, 4% the antenatal exercise classes, and 1% (*n* = 2) said “other methods”. The majority of mothers followed five sessions [min = 1-max = 10]. No significant associations between the number of attended sessions and the satisfaction with or perceived usefulness of those sessions were found, except for the usefulness of the session about pregnancy. Mothers who followed only one session found the information about pregnancy significantly less helpful (Table 2). Data collection occurred between 2 to 29 months after birth. There were no significant associations between satisfaction with perceived usefulness of the AC and the time between birth and data collection (Table 2).

**Table 2:** Relations between satisfaction or usefulness of AC, completion time or number of sessions attended

M ± SD (*n* = 289)	Between 0 to 6 months after the birth (*n* = 12)	Up to 12 months after birth (*n* = 83)	Up to 18 months after birth (*n* = 60)	Up to 24 months after birth (*n* = 61)	More than 24 months after birth (*n* = 43)	F (Anova)	*p*
Overall satisfaction	3.58 ± 0.79	3.61 ± 0.96	3.8 ± 0.88	3.49 ± 0.91	3.23 ± 1.15	2.34	.055
Overall usefulness	3.75 ± 0.75	3.62 ± 0.93	3.52 ± 0.99	3.33 ± 0.94	3.14 ± 1.15	2.35	.054
Usefulness regarding pregnancy	3.25 ± 1.06	3.10 ± 1.09	3.00 ± 1.07	2.81 ± 1.03	2.88 ± 1.20	.85	.85
Usefulness regarding childbirth	3.33 ± 1.07	3.38 ± 1.07	3.02 ± 1.2	3.12 ± 1.07	2.88 ± 1.33	1.67	.16
Usefulness regarding baby’s needs	2.67 ± 1.15	2.59 ± 1.22	2.80 ± 1.25	2.41 ± 1.12	2.41 ± 1.24	.98	.42
Usefulness regarding mothers’ needs	2.2 ± 1.64	2,87 ± 1.70	2.79 ± 1,68	3.32 ± 1.52	2.98 ± 1.60	.87	.49
	One session (*n* = 50)	2 to 4 sessions (*n* = 47)	More than 4 sessions (*n* = 130)			F (Anova)	*p*
Overall satisfaction	3.60 ± 1.05	3.62 ± 0.95	3.51 ± 0.92			.31	.73
Overall usefulness	3.28 ± 1.01	3.51 ± 1.00	3.45 ± 0.95			.95	.39
Usefulness regarding pregnancy	**2.46 ± 1.15**	**3,09 ± 0.95**	**3.05 ± 1.04**			**6.19**	**.002**
Usefulness regarding childbirth	3.08 ± 1.18	3.23 ± 1.11	3.11 ± 1.14			.25	.78
Usefulness regarding baby’s needs	2.55 ± 1.3	2,57 ± 1.28	2.50 ± 1.18			.07	.93
Usefulness regarding mothers’ needs	2.63 ± 1.61	3.19 ± 1.63	3.18 ± 1.53			.96	.39

*M* Mean, *SD* Standard deviation

Depending on the questions, between 91 and 138 mothers made comments used in the thematic analysis. Detailed information about characteristics of the mothers whose quotes are included in the text are available as supplementary material (Table S2). From the five main domains, the data-driven inductive approach made it possible to highlight three main themes and nine sub-themes (Table 3). The three main themes were: “1. Satisfaction and usefulness of classes”, “2. Timing of classes”, and “3. Content of classes”.

**Table 3:** Domains, themes, subthemes, and example quotes emerging from the qualitative data analysis

Domains: overall satisfaction/usefulness; satisfaction/usefulness linked to pregnancy, childbirth, newborn’s and mother’s needs		
Themes	Sub-themes	Example of verbatim
Satisfaction and usefulness of classes	Peer support	“I enjoyed the contact with the other parents” (P371) “The group of parents was very nice and we were able to share a lot of things. It was also great to meet up again after the childbirth.” (P417) “We failed to connect with the other parents, most of whom annoyed us.” (P911) “Meet other future parents with the same questions” (P1174) “However, it is interesting to know that as young parents we generally have the same issues/questions about parenthood.” (P1672)
	Psychological well-being	“The fact that my questions were answered reassured me” (P151) “This allowed us to project ourselves, to answer questions that were still a bit stressful and the visit to the CHUV also helped to reassure us (knowing where to go, who to contact, delivery room).” (P995) “Especially the fact that you shouldn’t plan your delivery. It rarely goes as planned The visit to the delivery room: it’s quite reassuring to have already been there” (P1006) “Discussing the subject of postpartum depression in its non-accusatory context was beneficial to me, it took away some of my stress and apprehension.” (P1936) “Exchanging with other people who are in the same situation as us is reassuring.” (P2090)
	Moving from teaching to practice	“My delivery didn’t go as well as I thought and I couldn’t put into practice what was taught” (P276) “Theoretical information on labour did not apply to me, constant pain, no pause between contractions due to [fetal] presentation” (P344) “On the moment of childbirth I did not put into practice what I had learnt” (P919) “Pain so intense that it is difficult to keep control of the body and apply the exercises.” (P2259)
Timing of classes	Timing of classes	“I would have preferred earlier in case I had an early delivery “ (P52) “Not much of a topic [baby’s care], but maybe it’s better that way. One thing after another, right?” (P208) “It’s not the time I was most receptive to after the birth.”(P64) “Too early for me but finally it was nice because I had to stop because of my edemas” (P608) “As long as the baby is not here, it is difficult to retain all the information.” (P819) “Maybe doing it earlier would have been less tiring (I’m talking for someone like me who worked full-time up to 37 weeks).” (P1531) “I didn’t learn much or it was too late to learn it. I would have liked to have the course around the 5th month” (P2320)
Content of classes	Information about stages of childbirth	“Vague notion of the stages of childbirth to know what to expect” (P2310) “Useful because we knew the different steps.” (P2214) “Lack of precision in some phases.—I was told that labour was inevitably long for a first one, I didn’t understand what was going on when everything went very fast.—no info on how to push correctly—no info on expulsion of placenta. I felt unprepared at the end of the birth.” (P2259)
	Coping with pain	“More advice on pain management would have been beneficial” (P15) “Breathing exercises have been a beneficial aid during the management of contraction-related pain” (P318) “The courses were very comprehensive and informative, but we did not see the breathing exercises and positions, or the possible physical interventions of the spouse, which might have been useful or relieved me” (P526) “Not enough exercises to explain pushing and breathing.” (P575)
	Protocols, hospital procedures and interventions	“I think the only useful information was “ if my water breaks, I go to the CHUV”. ” (P32) “Practically no mention of the provocation of the childbirth and therefore not informed of the procedure” (P233) “When I lost red blood, I knew right away that this was not normal…” (P1998) “Mention when to call the CHUV (first contractions, frequency, pain etc.).” (P1264)
	The unexpected/unnatural childbirth	“Unfortunately I had to have a caesarean section. I didn’t feel prepared for it and everything that followed” (P52) “It was good but I don’t think there is enough talk about the complications that can arise, especially about why a C-section has to happen. I always thought, in my ignorance, that they only happen if the baby is breech. This should be talked about more.” (P608) “The childbirth did not occur as the standard stated in the sessions.” (P609)
	Postnatal care (mothers and babies)	“Nothing about the postnatal period, when you are much more alone, when you have questions about pain, physical disabilities, etc.” (P20) “No explanation of baby care as putting a baby in a car seat for example. It’s too parenting based. No practice offered” (P52) “I have the impression that there is never enough information on breastfeeding (although it is difficult), we don’t know a lot about the losses we are going to have, the perineum rehabilitation, etc.”(P64) “talk more about the existential upheaval that parenthood involves” (P276) “Not enough content on risks when returning home (depression, isolation, sudden death etc.)” (P456) “Nothing was covered in these courses and I think it’s a shame because I didn’t know how to deal with a child.” (P575) “We didn’t talk much about the baby’s first few weeks. Everything we were able to discuss was useful. We also learned about the resources and professionals available to us for this stage (suggestion of the midwife after the birth, breastfeeding help, etc.).” (P1249) “I think that sleep (parent and child) is an important point, mentioning the evolution over the first 6 months (the child must learn to fall asleep alone, techniques etc.)” (P264) P 2340 would have liked to discuss "Postpartum depression, hormone crash, fatigue and normalization of emotions of a woman who has just given birth"

### Satisfaction and usefulness of classes

More than half of the mothers (61.0%; *n* = 158) were very or extremely satisfied with AC and 56.2% (*n* = 145) found these sessions very or extremely helpful. The timing of the sessions appeared mostly appropriate (83.6%; *n* = 209), although too late for 12.8% (*n* = 37) of mothers. Moreover, 85.9% (*n* = 221) of the mothers who attended AC would recommend these sessions to a friend.

One of the main sources of satisfaction was peer support. It is the first most useful point of AC cited by mothers (17.6%) not related to childbirth (see Fig. 3). The group allowed sharing *“feelings and worries”* (P84), experiences and concerns (P414) “*with other people who are in the same situation”* (P661)*,* which was seen as *“reassuring”* (P661) and *“very precious”* (P1936).

Regarding usefulness of AC, the best-rated session was the childbirth session, found to be very or extremely helpful by 43.9% (*n* = 108) of mothers. Satisfied mothers stated that AC provided clear information, allowed feeling more secure, confident and well prepared for the childbirth, even in the case of difficulties and complication. The midwife helped them to *“feel confident”* (P320) and the fact that mothers can “*ask the midwife any questions about the birth and the pregnancy*” led to a feeling of being accompanied (P390). Breathing exercises were mentioned as “*a beneficial aid during the management of contraction-related pain*” (P94). However, some mothers also reported difficulties in moving from teaching to practice *“Because when it comes to the real labour, the real pain, the adrenaline, the emotion, *etc*., we forget everything we have learned during these courses!”* (P481).

**Fig. 2:**
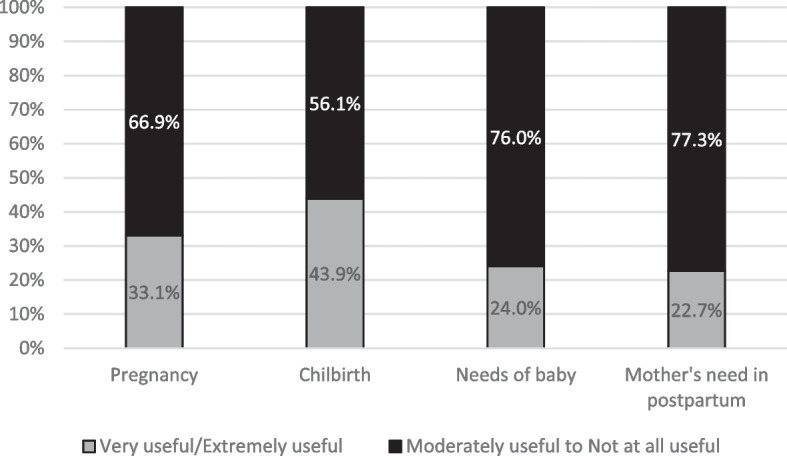
Participants’ perceived usefulness ratings according to the different topics addressed during antenatal classes (*n* = 289)

However, no session was overwhelmingly rated as very or extremely useful (Fig. 2).

Only nine mothers (3%), rated each session as very or extremely helpful. The least useful session was the one on the needs of postpartum mothers, which was considered very or extremely useful by only 22.7% of them (Fig. 2). Information about postnatal care for mothers and newborns was found to be globally of little use, with about 47% (*n* = 55) of the mothers saying that this subject was poorly discussed. Newborn’s needs were not specifically discussed, and this was felt by a participant as *“a shame because* [she] *didn’t knows how to deal with a child.”* (P185). Regarding mothers’ needs, the lack of information and dialogue on these issues gave participants the impression that *“the postpartum period, which takes place at home in the following days, remains taboo”* (P594). Moreover, the lack of solutions to prevent difficulties was also a source of dissatisfaction: “*We hear about potential postpartum depression, but how can we avoid it? What are the keys to a successful postpartum? What can we do before the baby arrives to make sure we feel good after giving birth?”* (P224).

### Timing of classes

Mothers found the timing of the sessions adequate (89%, *n* = 199), but questioned the timing of some of them in the comments. For instance, the session about pregnancy *“take place close to the end of the pregnancy”* (P481) and *“there was little connection with (…)* (the) *pregnancy”* (P268). Mothers felt “*already familiar with the course of the pregnancy”* (P481). The question of the timing of sessions also arose with regard to sessions dedicated to mothers’ and newborns’ needs. Some mothers felt that discussing postnatal care in AC “*was not the purpose.”* (P145). It was *“Too early to think about it almost”* (P219) and it seems better to advance *“step by step on these points”* (P280). Perhaps, mothers would be *“most receptive to this after the birth”* (P29) as *“as long as the baby is not here, it is difficult to retain all this information”* (P266)*.* Finally, the AC may not be the best place to discuss this but “*rather with the midwife who visits at home”* (P280) because *“The change of life is so radical that the courses cannot prepare* (them) *for it”* (P439). Finally, a woman raised the question of the place of post-natal support:

*“I never imagined that the hormonal drop would be so difficult to live with, the feeling of emptiness left inside me, the fusion that I miss with my baby, the doubts, the fatigue, dealing with the people around me... (…) It would be very useful to be able to open a dialogue on this point and to warn future mothers of this important challenge...”* (P30).

### Content of classes

**Fig. 3:**
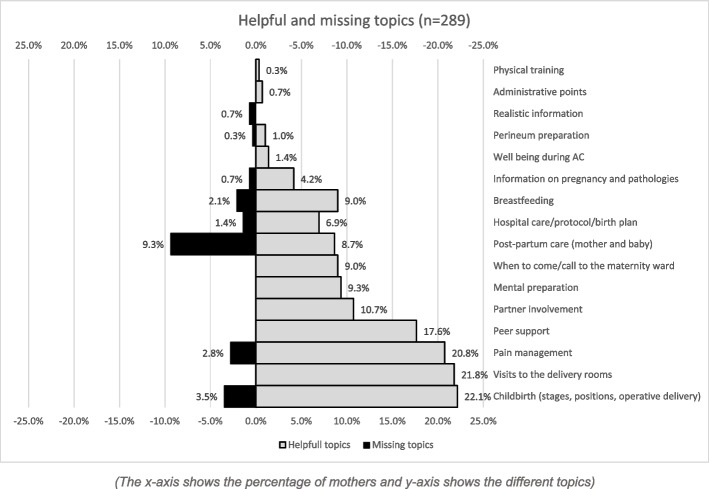
Percentages of mothers perceiving topics discussed during the antenatal classes as helpful or missing

The conceptual content analysis revealed that the three most helpful topics were related to childbirth: stages of childbirth including duration of labour and birthing positions, the visits of the delivery rooms and the pain management including breathing, relaxation, and positioning during contraction to alleviate pain (Fig. 3).

Explanations about the stages of childbirth were seen as helpful *“to de-stress*” (P729) and “*knowing when to call the hospital (first contractions, frequency, pain, *etc*.)* was reassuring (P396). Mothers particularly highlighted “*some useful exercises to relieve the contractions (…)”* (P396) and the “*provision of useful ways to manage pain, concentration, and breathing*” (P820) to *“feel empowered during the birth”* (P390). Moreover, these sessions also provided the opportunity to explain the hospital procedure “*The arrival protocol, explanations of possible medical procedures, the reasons for them, and how they are carried out”* (P4). Finally, the sessions provided a space to involve the partner in the process of pregnancy and childbirth and for the postnatal, thus relieving the woman of the teaching role towards her partner: Finally, *“The sessions strengthened the ‘team’ spirit in the couple.”* (P390). One woman summarised particularly well this topic saying: *“I was afraid of childbirth, so having the right information helped me. To know when labour started when to go to the maternity ward, the different positions to reduce the pain of contractions and, above all, to involve my partner in this process*” (P201).

However, the majority of mothers were dissatisfied with the content of the different sessions that they found incomplete and/or inadequate. For example, among the mothers who responded that childbirth classes were moderately to not at all helpful, 19% (*n* = 13) had a caesarean section and received no information on this topic. Dissatisfaction of mothers came from a feeling that the midwife avoided talking about possible difficulties and complications. The content of the sessions seemed to be too centred on natural birth and mothers felt not prepared for other situations. A woman said, *“We are not told everything to “protect” us and in the end, we feel a bit lost when the day comes.”* (P 276), while another highlighted “*a tendency to want to show the hunky-dory side of things”*, which leads to feeling “*so disappointed afterwards when there are complications”* (P454).

Finally, one woman expressed the feeling of “*frustration”* about the birth plan developed with the help of AC and thought that “*Preparing to ‘welcome’ the unexpected would have been more useful.”* (P382). Mothers suggested that “*complications need to be addressed clearly and mothers need to be told what can happen in certain situations, so that they can react or interact in the best possible way during the birth*.” (P454). Dissatisfied mothers found in the sessions “*A lot of banalities, lack of dialogue, little information on a less conventional delivery (e.g., caesarean section), very little information on postpartum in general, and very little information on breastfeeding”* (P516), with also “*little information on pregnancy in general and on the stages of development of the baby in the womb”* (P460). Moreover, *“the sessions only repeat the content of the received documents”* (P11) and as *“the internet already provides a lot of information”* (P198) it makes *“the preparation sessions (…) boring…”(*P297). Furthermore, some mothers questioned the correspondence of the content of the sessions with the reality of childbirth: “*The contractions didn’t go as I had imagined. We realised that it wouldn’t be like what we had prepared for*.” (P126).

Regarding the postpartum, the great majority (76%) of the mothers found the content of the sessions useless. The conceptual content analysis showed that the most frequently mentioned missing topic was the postnatal including the return home and the impact on lifestyle (see Fig. 3)*.* For some mothers*, “postpartum needs were extensively discussed. Lots of info were given about where we could find support/help in case of postpartum-related issues” (P84).* Moreover, *“hearing some advice allowed to talk about it as a couple and to prepare, to take some decisions” (P126).* Finally, *“the description of the possible postpartum states was reassuring afterwards when fatigue and panic overtook us. You don’t feel alone” (P820).* Finally, the presence of the partner during this session was considered helpful in order to support the childbearing mother: *“because otherwise I would have totally forgotten myself for the baby. My husband had learned from the course what my needs would be and he looked after me while I looked after the baby.” (P725).* However, the majority considered that *“this is a big gap in general” (P330), with “little mention of the baby blues. No mention at all about vaginal bleeding, soreness, breast tenderness” (P369).* They also wanted *“more info about postpartum depression risks and recovery from childbirth” (P464).* Opinions about breastfeeding were more contrasted. Half of the mothers found this session *“informative, warm, and professional” (P 714), “useful and necessary (P313*) and especially useful *“to understand that breastfeeding takes time… there, the numbers, days, averages…”(P 611)* with solutions to deal with breastfeeding (P842). The other half were not satisfied with these sessions, as they found them *“very quick and rather superficial”* (P594) with a kind of *“idealisation of breastfeeding” (P11).* Moreover, some mothers were very dissatisfied to experience *“a lot of pressure on breastfeeding”* (P 483) and one of them felt the need to *“prepare (herself) for the painful pro-breastfeeding propaganda that awaited (her)” (P 603).* Moreover*, “no info on bottles, although (they) would have deserved to have this info too.” (P 483)* and *“some information on infant colic would have been useful” (P712).* Mothers also noted *“few explanations on the first days of life (of the baby), i.e., the most destabilizing, sleep, breastfeeding” (P372).*

## Discussion

This mixed-method study investigated satisfaction and usefulness of AC after the birth which is, to our knowledge, the first time this was done in Switzerland. The quantitative part of the study found that less than two-third of mothers after birth were satisfied with the AC. In the qualitative part, mothers questioned the usefulness of these sessions, their content, as well as the timing of some of these sessions. They wanted to be more informed, in a realistic way, and not to be overprotected regarding possible risks or complications of childbirth and postpartum.

One important point our participants raised was that AC were an important space for sharing and meeting other future parents. This is in line with previous research showing that AC improved peer support [24] and were a source of knowledge [25]. Moreover, sharing problems with other parents normalizes their concerns [25].

The lack of perceived usefulness of AC may partly be explained by the fact that data collection occurred after the birth. Another study showed that comments about the content of these sessions were less favourable when evaluated after the birth rather than after the completion of AC [26]. However, mothers in an Italian study described the AC as very useful when asked after the birth even if, as in our study, the ability to put into practice the knowledge of the sessions was the more difficult thing [14]. Yet, the number of sessions and the stakeholders were different. Moreover, the completion of the questionnaire occurred in the immediate postpartum period, which could explain some of the differences in the results, especially with regard to satisfaction with the postpartum period and care of the baby [14].

Regarding the timing of the sessions, others studies have already highlighted that the pregnancy session arrived often too late and recommended starting AC in the second trimester [27] or, according to parents’ opinions, before conception or at the beginning of pregnancy [28]. Breastfeeding and its potential problems seemed to be, for the parent, the most important point to be addressed during AC [29]. However, this theme, together with the topic of parenting, was addressed at the very end of pregnancy, after the childbirth session, while mothers seemed to be interested in motherhood, and particularly in breastfeeding, from the 24^th^ week of pregnancy [30]. Allowing parents to discuss this information earlier in the pregnancy, at a more privileged moment of attention, might be preferable. Finally, parents would also prefer sessions to continue up to one year after birth in order to discuss more about parenting [30].

Regarding the content, one of the top two reasons for AC attendance was to feel more secure when taking care of their newborn [31]. However, this theme seemed to be poorly discussed in our study, even if 40% of the antenatal sessions were devoted to the postnatal. This was also the case in other studies [27, 32], with up to 67% of the content being relative to childbirth [27]. In a Swedish study, only 40% of mothers thought that AC helped them prepare for early parenthood [33]. This feeling of unpreparedness for parenthood was widely shared by parents in the literature, even though some mothers thought that they might have forgotten some AC information [32]. Regarding breastfeeding education, mothers felt largely unprepared for potential breastfeeding complications [16, 26, 27], as half the comments in our study showed. Midwives are particularly convinced of the value of breastfeeding and actively transmit this message [34]. The discourse is sometimes perceived to be more coercive than informative [34], something that some of our mothers also seem to have experienced.

### Strengths and limitations

One of the main strengths of our study is that comments were collected after the birth. This enabled the mothers to better evaluate the usefulness of the information provided, to realise what information was missing, as well as the difficulties in putting them into practice. Providing questionnaires in French and English was also a positive point, as this covered the two main languages used in the hospital providing the AC.

However, this study has a number of limitations. First, some of the mothers may have forgotten some points explained in the sessions, as for some participants, up to 2 years had passed since birth. In addition, some women may have become pregnant again between the first birth and the time of data collection. Furthermore, mothers may have only retained the most salient points of their experience. Additionally, the low response rate of the main study (27.6%) means that the participants’ remarks can be seen as a non-exhaustive reflection of what mothers think about prenatal care, although the response rate is comparable to other questionnaire studies [35–37], particularly to online surveys [38, 39]. Our sample only represents a relatively privileged population compared to the general population, even though it is typical for the population that attends AC in this hospital. Moreover, the low caesarean rate of the sample (21.9%) compared to the caesarean rate of the hospital at this time (25%) suggests that our sample is not fully representative of the population of women giving birth in a Swiss hospital. The sample over-represented women who had a partner or were married (69.1% in our study versus 49% in the canton of Vaud), as well as women with a high level of education (71% in our study versus 42% in the canton of Vaud). Finally, this study was only conducted in one Swiss university hospital, thus limiting generalization of the results.

### Implications for research and practice

As the AC do not meet the needs of mothers, it seems necessary to investigate their needs and, broadly, the needs of couples in terms of preparation for childbirth and parenthood, and to see if common needs exist across countries. A prospective approach would allow to reach all the women who attended antenatal classes and to compare responders and non-responders, and is therefore recommended. Furthermore, future studies should consider the use of a validated tool in order to measure satisfaction. Based on these needs, a new model of AC will have to be developed, guided by adult learning principles [40, 41]. Moreover, this new model should take into consideration mothers with a lower level of education and less financial means, who are often excluded from AC, even if the sessions are free of charge [18, 42]. Finally, standardisation of the basic content and training of those involved will also be necessary to generalise this new AC approach [7, 43, 44]. In parallel, it seems necessary to systematically evaluate this.

## Conclusion

Mothers were mostly satisfied with AC but asked for a realistic view of childbirth and a better preparation for the postpartum period. Therefore, this study showed that it is necessary to rethink and adapt AC based on service users’ needs.

### Supplementary Information

**Additional file 1: Supplementary materials 3.**

**Additional file 2: Table S1.** Brief description of antenatal sessions (scenarios are available in French on request to the authors).

**Additional file 3: Table S2.** Obstetrical and neonatal outcomes of mothers whose citation are include in the text.

## Data Availability

The datasets used and/or analysed during the current study are available from the corresponding author on reasonable request.
