# Supplementary material for: What do mothers think about their antenatal classes? A mixed-method study in Switzerland
Source: BMC Pregnancy Childbirth. 2023 Oct 19;23:741. doi: 10.1186/s12884-023-06049-8 (PMC10585766; doi:10.1186/s12884-023-06049-8)
Supplement: Supplementary file 1 — Additional file 1: Supplementary materials 3. [file 12884_2023_6049_MOESM1_ESM.pdf]

Supplementary materials 3

Satisfaction of birth preparation at the CHUV

The questions below refer to the preparation for birth and parenthood course at the CHUV (Lausanne University Hospital). Please reply only if you have attended these sessions.

Surround the face that corresponds best to answers the questions below.

1. What is your overall satisfaction with the preparation for birth and parenthood course at the CHUV? Please encircle appropriate face.

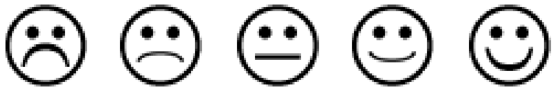

Not at all satisfied	A little bit satisfied	Moderatly satisfied	Very satisfied	Extremly satisfied

Specify :

2. In general, to what extent did you find these classes useful? Please encircle appropriate face.

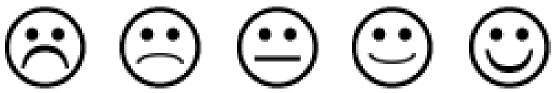

Not at all useful	A little bit useful	Moderatly useful	Very useful	Extremly useful

Why ?

3. To what extent did you find these classes useful during your pregnancy? Please encircle appropriate face.

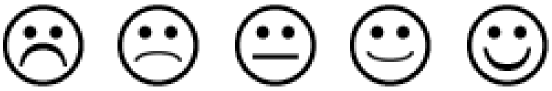

Not at all useful	A little bit useful	Moderatly useful	Very useful	Extremly useful

Why ? ....

....

....

....

....

4. To what extent did you find these classes useful for the actual labour and childbirth? Please encircle appropriate face.

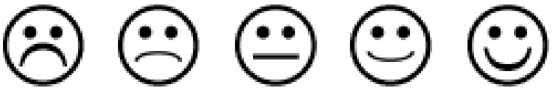

Not at all useful	A little bit useful	Moderatly useful	Very useful	Extremly useful

Why ? ....

....

....

....

....

5. To what extent do you find these classes useful to understand your baby's needs (feeding, sleep, affection, hygiene ...)? Please encircle appropriate face.

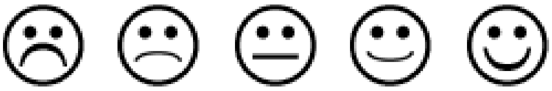

Not at all useful	A little bit useful	Moderatly useful	Very useful	Extremly useful

Why ? ....

....

....

....

....

6. To what extent do you find these classes helpful in understanding your own postpartum needs? Please encircle appropriate face.

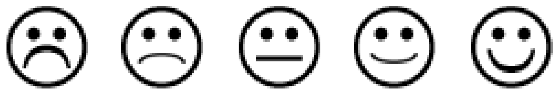

Not at all useful	A little bit useful	Moderatly useful	Very useful	Extremly useful

Why? ....

....

....

....

....

7. Would you recommend these antenatal classes to a friend? Surround appropriate.

YES **NO**

8. What were the three most helpful things with regards to the course?

a. ....

....

b. ....

....

c. ....

....

Comments: ....

....

....

9. What was the most difficult for you during these classes?

....

....

....

....

....

10. Has the timing of the prenatal classes suited you? Please encircle appropriate words

Too earlier **Just in time** **Too late**

Comments : ....

....

....

....

....

11. Is there anything you would have liked to discuss during antenatal classes that was not discussed?

....

....

....

Other comments :

....

....

....

Thank you for your participation
