# Supplementary material for: What do mothers think about their antenatal classes? A mixed-method study in Switzerland
Source: BMC Pregnancy Childbirth. 2023 Oct 19;23:741. doi: 10.1186/s12884-023-06049-8 (PMC10585766; doi:10.1186/s12884-023-06049-8)
Supplement: Supplementary file 2 — Additional file 2: Table S1. Brief description of antenatal sessions (scenarios are available in French on request to the authors). [file 12884_2023_6049_MOESM2_ESM.pdf]

Supplementary materials

Table S1: Brief description of antenatal sessions (scenarios are available in French on request to the authors)

Sessions	Themes	Modalities	The essentials	Body workshop - 30 min
1	**Pregnancy experience**	• No scenario/ Start from the experience of the couples<br>• "Tell us about it " -Work on representations<br>• -"How do you do it?" Work on coping strategies	Anatomical-physiological reminder/ Body and psychological changes/ Rythm of life- Work/ Rights of the pregnant and breastfeeding woman/ Reasons for emergency consultations/ Important telephone numbers/ Relationship with the child/ Optimising the couple dynamic/ Sexuality and pregnancy/ Resources	Pregnancy comfort exercises:<br>• Posture<br>• Breathing<br>• Relaxation<br>• Connection to the child
2	Childbirth<br>• Term of the pregnancy<br>• **Initiation of labour**	Using scenario N°1, help the couples to project themselves into the situation<br>• Work on representations<br>• How do you imagine yourself doing? Work on coping strategies	Duration of the pregnancy / Exceeding the presumed term / Induction of labour / Contractions: managing the pain / At home / When to go to the maternity hospital / How to live it as a couple	• Breathing<br>• Relaxation :<br>•Relaxation<br>•Massage<br>•Positions<br>• Support
3	Childbirth:<br>• Pain<br>• Duration<br>• Birth<br>• - First contact with the child	Using scenario No. 2, help the couples to project themselves into the situation<br>• Work on representations<br>• How do you imagine yourself doing? Work on coping strategies<br>• Visit to the delivery room (on site/virtual)<br>• Visit to the postnatal ward	Mechanism/ Letting go/ Ways of managing/supporting pain/ Preventing exhaustion/discouragement/ Role of the companion/spouse/ Phases of VBAC (vaginal delivery) / Management in the delivery room/ Delivery positions (VBAC) / Perineum / Birth aids (forceps, vacuum) / Elective/emergency caesarean section/ Expulsion/delivery/ Welcoming the newborn during VBAC/caesarean section/ Reasons for separation from the newborn	• Breathing<br>• Relaxation :<br>•Relaxation<br>•Massage<br>•Positions<br>• Support<br>• Experimenting with reactions to discomfort<br>• Testing birthing positions<br>• Testing different forms of push-ups and positions
4	Staying in the maternity ward<br>• First days after delivery<br>• Needs of the newborn<br>• - Breastfeeding	Using scenario N°3, help the couples to project themselves into the situation<br>• Work on representations<br>• How do you imagine yourself doing? Work on coping strategies	Postnatal care/ Length of stay in maternity hospital/ Anticipation of return home/ Presence of spouse/ Perineum (reeducation)/ Fatigue/ Pain/ Baby blues/ Role of caregivers/ Physical and emotional safety/ Sleep/wake rhythm/ Crying/ Feeding/ Deviations from norm/ Choice of feeding method/ Physiology of lactation/ Support/ Means/ Artificial feeding/ Deviations from norm	• Tightening of the pelvis<br>• Posture
5	Going home - becoming parents<br>• Organisation at home<br>• Social support	Using scenario N°4, help the couples to project themselves into the situation<br>• Work on representations<br>• How do you imagine yourself doing? Work on coping strategies	Change of role / Taking care of oneself / Taking care of one's child (SIDS, shaken baby) / Taking care of one's couple (communication, sexuality, contraception) / Preventing exhaustion, isolation / Screening for postnatal depression / Building the support network: family, friends and professional circle / Resuming work	• Internalization/stress experience<br>• Breathing<br>• Relaxation<br>• Massage
6	Postnatal<br>• Organisation at home<br>• Relationship to the child<br>• Relationship to the body<br>• Child's nutrition	• No scenario/ Start from the experience of the couples<br>• "Tell us about it " -Work on representations<br>• "How do you do it?" Work on coping strategies	Taking care of yourself/your child/your couple/ Parenting process/ Communicating/ Going back to work/ Detecting difficulties and looking for resources	
