# Supplementary material for: What do mothers think about their antenatal classes? A mixed-method study in Switzerland
Source: BMC Pregnancy Childbirth. 2023 Oct 19;23:741. doi: 10.1186/s12884-023-06049-8 (PMC10585766; doi:10.1186/s12884-023-06049-8)
Supplement: Supplementary file 3 — Additional file 3: Table S2. Obstetrical and neonatal outcomes of mothers whose citation are include in the text. [file 12884_2023_6049_MOESM3_ESM.pdf]

Supplementary materials

Table S2: Obstetrical and neonatal outcomes of mothers whose citation are include in the text

N° of the participant (inclusion in RedCap)	Mode of childbirth	Type of anaesthesia	Hospitalisation of baby in NICU	Number of antenatal sessions followed
4	spontaneous vaginal delivery	epidural	no	4
8	spontaneous vaginal delivery	epidural	no	6
11	forceps	epidural	no	1
15	spontaneous vaginal delivery	epidural	no	6
20	forceps	epidural	no	1
29	spontaneous vaginal delivery	local	no	4
30	spontaneous vaginal delivery	epidural	no	NA
32	spontaneous vaginal delivery	epidural	no	1
52	emergency cesarean	Spinal	no	5
64	spontaneous vaginal delivery	local	no	4
84	emergency cesarean	general	no	NA
94	forceps	epidural	no	6
103	emergency cesarean	epidural	no	5
111	spontaneous vaginal delivery	local	no	NA
125	emergency cesarean	spinal	no	5
126	spontaneous vaginal delivery	epidural	yes	5
141	emergency cesarean	epidural	no	6
145	vacuum extraction	epidural	yes	1
151	spontaneous vaginal delivery	local	no	4
185	spontaneous vaginal delivery	epidural	no	4
198	spontaneous vaginal delivery	none	no	4
201	spontaneous vaginal delivery	epidural	no	5
208	emergency cesarean	epidural	no	1
212	vacuum extraction	epidural	no	5
219	spontaneous vaginal delivery	local	no	4
224	emergency cesarean	spinal	yes	2
233	emergency cesarean	epidural	yes	NA
266	forceps	epidural	no	5
268	spontaneous vaginal delivery	epidural	no	6
276	emergency cesarean	spinal	no	6
280	spontaneous vaginal delivery	local	no	4
297	spontaneous vaginal delivery	local	no	6
313	spontaneous vaginal delivery	local	no	1

318	forceps	epidural	no	6
320	emergency cesarean	spinal	no	NA
330	spontaneous vaginal delivery	local	no	4
344	emergency cesarean	epidural	no	5
369	spontaneous vaginal delivery	epidural	yes	5
371	emergency cesarean emergenc	epidural	yes	5
372	emergency cesarean	epidural	no	5
378	emergency cesarean	spinal	no	5
382	vacuum extraction	epidural	no	7
390	spontaneous vaginal delivery	none	no	5
396	spontaneous vaginal delivery	local	no	5
397	forceps	epidural	no	5
414	spontaneous vaginal delivery	spinal	no	5
417	emergency cesarean	epidural	no	5
439	emergency cesarean	spinal	no	4
454	forceps	epidural	no	1
456	Elective cesarean	spinal	no	10
460	spontaneous vaginal delivery	spinal	no	1
464	spontaneous vaginal delivery	epidural	no	5
481	spontaneous vaginal delivery	epidural	no	1
483	spontaneous vaginal delivery	epidural	no	1
516	elective cesarean	spinal	no	6
526	spontaneous vaginal delivery	epidural	no	5
575	spontaneous vaginal delivery	epidural	no	4
594	forceps	epidural	no	NA
603	emergency cesarean	general	no	1
608	emergency cesarean	spinal	yes	5
609	Vacuum extraction	epidural	No	5
611	spontaneous vaginal delivery	epidural	no	5
661	spontaneous vaginal delivery	epidural	no	4
703	elective cesarean	spinal	no	6
712	spontaneous vaginal delivery	epidural	no	5
714	spontaneous vaginal delivery	epidural	no	2
725	emergency cesarean	epidural	no	5
729	spontaneous vaginal delivery	epidural	no	1
801	spontaneous vaginal delivery	local	no	6
805	spontaneous vaginal delivery	epidural	no	6

819	forceps	Epidural	No	5
820	spontaneous vaginal delivery	epidural	no	6
842	spontaneous vaginal delivery	local	no	5
911	spontaneous vaginal delivery	local	no	6
919	spontaneous vaginal delivery	epidural	no	5
995	emergency cesarean	spinal	no	NA
1006	spontaneous vaginal delivery	epidural	no	5
1070	spontaneous vaginal delivery	local	no	6
1174	spontaneous vaginal delivery	epidural	no	1
1249	spontaneous vaginal delivery	none	no	5
1264	spontaneous vaginal delivery	local	no	5
1304	spontaneous vaginal delivery	epidural	yes	4
1383	emergency cesarean	spinal	no	4
1531	spontaneous vaginal delivery	epidural	no	1
1672	spontaneous vaginal delivery	local	no	10
1846	forceps	epidural	no	10
1936	spontaneous vaginal delivery	epidural	no	5
1998	spontaneous vaginal delivery	epidural	no	NA
2090	spontaneous vaginal delivery	epidural	no	4
2214	spontaneous vaginal delivery	epidural	no	5
2259	Vacuum extraction	epidural	no	1
2310	Vacuum extraction	epidural	no	NA
2320	Vacuum extraction	epidural	yes	5
2340	Elective caesarean	Spinal	No	1

NA: not available
